# Beyond the Trauma Triad of Death—New Advances in Our Knowledge of Pathophysiology as a Basis for New Perspectives in Support Therapy

**DOI:** 10.3390/life12030428

**Published:** 2022-03-16

**Authors:** Giuseppe Ietto, Caterina Franchi, Gabriele Soldini, Francesco Amico

**Affiliations:** 1 General, Emergency and Transplant Surgery Department, ASST-Settelaghi, University of Insubria, 21100 Varese, Italy; franchicaterina88@gmail.com; 2 General and Emergency Surgery Unit, Holy Family Hospital Fatebenefratelli, 22036 Erba, Italy; dott.g.soldini@gmail.com; 3 Trauma Service, Department of Surgery, University of Newcastle, Newcastle 2308, Australia; francesco.amico@hotmail.com

The history of staged laparotomy, the basic idea behind the so called “Damage Control Surgery (DCS)” attitude, leans on the experience developed during the treatment of major hepatic trauma that was sustained by Pringle’s pioneering attempts to achieve hemostasis on liver wounds via packing in 1908 [1,2,3]. Overcoming the initial difficulties that seemed to contraindicate this staged approach for necrotic and septic complications on injured liver parenchyma, DCS has progressively gained more and more enthusiasm since 1976 [2].

The pathomechanistic insights on which the successes of this attitude are based is the identification of refractory coagulopathy as the impasse to patient survival. The “bloody vicious cycle” that is sustained and exacerbated by unattended core hypothermia and persistent metabolic acidosis are key events that promote this lethal coagulopathic state [3].

Modern knowledge regarding the management of major trauma clearly suggests that outcome is correlated with both the appropriateness and timeliness of diagnosis and treatment and that they are able to stop the “haemorrhage—coagulopathy vicious cycle”, but, first and foremost, this is dependent on the severity of the injury [4]. In fact, the extremely broad range of symptomatology developed after trauma as well as during the course of every significant severe acute disease seems to be correlated with the severity of the initial tissue damage and with the intensity through which the immune system reacts to this.

In fact, a severe trauma almost instantaneously leads to a high level of cell death due to the release of debris cell fragments. These DAMPs (damage-associated molecular patterns) sustain the following cascade and the pathophysiological consequences, which are structured in the mechanistic insight depicted in the so-called “Danger model” [4,5,6]. The “Danger model”, a paradigm described by Matzinger in 2002, suggests that the damage products and alarm signals from injured tissues are the main ones that are responsible for the activation of the immune system [7,8,9]. In addition to dangerous molecules, neutrophil-associated extracellular trap (NET) formation also plays an emerging role in sustaining inflammatory response [10,11,12,13,14].

Interestingly, according to the severity of the injury, the magnitude and the duration of this physiological derangement differ and will become responsible for the development of multiple organ dysfunction syndrome (MODS) as a result of excessive pro-inflammatory response (systemic inflammatory response syndrome (SIRS)) followed temporally by compensatory anti-inflammatory response syndrome (CARS) and the suppression of adaptive immunity [15,16,17]. The predominant presentation depends on the underlying prevalent pathomechanism: thrombotic microangiopathies and coagulopathies and/or the hyper activation of complement pathways that may lead to disseminated microvascular thrombosis; pathologic immune activation and hyper inflammation; immune paralysis with associated secondary bacterial or fungal infections; and an inability to combat inflammation due to CD4+, CD8+, and a deficiency in the number of dendritic cells.

The pathomechanisms are consistent with a genomic storm that is neither chaotic nor erratic, but that is rather highly coordinated and reproducible. This storm likely represents a common transcriptional response to severe stress in humans regardless of its origin, with far more similarities than differences [18]. A deep knowledge of these pathomechanisms might be extremely interesting in both predicting the outcome and defining the most therapeutically suitable approach after severe trauma.

In order to reduce the stress response and the following immune suppression after trauma, it is quite clear that minimizing infection is not only the result of using an adequate antibiotics prophylaxis and that the optimization of the immune response by maintaining homeostasis with nutritional support (especially enteral) is fundamental [19]. To this end, reducing every possible additional trauma due to any operative post-injury management which would be the result of surgery) also plays a pivotal role. In the light of this overview, the basic principle that would have to act as a beacon light for any efficacious treatment after trauma is to resolve the injury that is limiting the further release of DAMPs.

From this perspective, a surgical procedure might be justified in order to obtain this goal whenever no other convenient opportunities are available, enabling a faster recovery. Every haemostatic procedure and surgery itself exacerbate cell death directly, as do the additional ischemic events that follow the manoeuvres required to stop the bleeding: the embolization and/or ligation of vessels. Whenever a resective procedure is not followed by hemostasis, the ischaemic tissues will be invaded by immune cells to clean and repair the necrotic area through regenerative and scarring processes. When too extensive, this immune activation could significantly contribute to the development of Systemic Inflammatory Response Syndrome. The outcome will depend on the damage to multiple organs caused by the initial cascade of inflammation aggravated by the subsequent sepsis to which severe injury is associated [6].

Many new studies have analysed therapies to prevent multiorgan failure and that are directed at the targets of the SIRS, and several treatment strategies have been targeted to reduce the cascade of events triggered by activated neutrophils. A large number of these therapies (e.g., prostaglandin E1, monoclonal antibodies against β2 intergrin, anti-L-selectin (CD62L), and leukocyte-reduced blood) targeting activated neutrophils have so far shown few results [19]. In contrast, two already well-known therapies have demonstrated real efficacy after trauma: hydrocortisone and tranexamic acid. In one RCT, systemic hydrocortisone was reported to halve hospital-acquired pneumonia and increase the number of mechanical ventilation-free days. Tranexamic acid, which inhibits fibrin degeneration, seems to contribute to improved survival not only by improving clot stability, but also by restricting the inflammatory response that can be provoked by fibrin degradation products [20].

The use of dehydroepiandrosterone (DHEA) seems to be promising for the treatment and rehabilitation of injured patients. Some studies, starting from the observations that being male and increasing age are associated with increased mortality in traumatic injuries, have postulated that there is a relationship with the sex steroid precursor hormone DHEA [21]. Cortisol levels are elevated after severe injury, as is sepsis. Glucocorticoids are potent s immune system suppressors and promote an intra-adrenal shift in steroid biosynthesis, which is produced at the expense of DHEA; indeed, the DHEA level is reduced in a few days after trauma and remains low for 3 months [22]. In DHEAS, the cortisol ratio is supposed to represent a balance between the catabolic effects of cortisol and the regenerative effects of DHEA, and its modulation may benefit the trauma patient. Animal models have demonstrated numerous benefits of DHEA supplementation, such as improved hyperglycaemia, decreased mortality after trauma-induced haemorrhage, neurogenesis, and wound reperfusion, all of which pose a considerable burden to injury recovery. Unfortunately, there are, for now, very few studies on the effects of DHEA on human immune cells. DHEA has been shown to directly stimulate the action of NADPH oxidase and reactive oxygen species production and thus improve neutrophil function [22]. Another benefit of DHEA supplementation has also been proposed: DHEA seems to have psychological and neurological effects and has been shown to promote neurogenesis in the hippocampus and the survival of newly formed neurons. Finally, several studies have suggested a positive effect of DHEA on bone biology; as traumatic injuries and hip fractures consist of characteristic long bone and soft tissue injuries, the potential effects of DHEA as a means of improving a patient’s bone profile in recovery may be advantageous.

In conclusion, when a trauma patient responds to resuscitation and does not deteriorate immediately, a less aggressive treatment is preferred. Whenever surgery becomes necessary, efforts should be made to limit the amount damage caused by surgery. As such, the progression of the damage will be counteracted, and the total number of DAMPs will be reduced. Additionally, adequate adjuvant treatment might be advisable to counteract the pathophysiological mechanisms of coagulopathy and the inflammatory response.
